# *Macleaya cordata* extract improves egg quality by altering gut health and microbiota in laying hens

**DOI:** 10.1016/j.psj.2024.104394

**Published:** 2024-10-10

**Authors:** Guoxin Zhang, Bochen Song, Xue Pan, Chake Keerqin, Okasha Hamada, Zhigang Song

**Affiliations:** ⁎ Shandong Provincial Key Laboratory of Animal Nutrition and Efficient Feeding, College of Animal Science and Technology, Shandong Agricultural University, Taian, Shandong 271018, China; † Hebei Key Lab of Laboratory Animal Science, Hebei Medical University, Shijiazhuang 50017, China; ‡ Phytobiotics (Jiangsu) Biotech Co. Ltd., Changzhou, Jiangsu 213200, China; § Animal Production Department, Faculty of Agriculture, Benha University, Moshtohor 13736, Egypt

**Keywords:** *Macleaya cordata* extract, Hyline brown laying hen, laying performance, immune function, intestinal microbiota

## Abstract

This study investigated the effect of *Macleaya cordata* extract (**MCE**) on the performance, gut health, and microbiota of laying hens. A total of 192 thirty-wk-old Hyline brown laying hens were randomly divided into 4 treatment groups. The CON group received a basal diet, while the low (MCE250), medium (MCE350), and high (MCE450) dose groups were supplemented with 250, 350, and 450 mg/kg MCE, respectively. The egg weight and Haugh unit demonstrated a linear and quadratic increase with the MCE dose during the initial 4-wk period of the experiment (*P* < 0.05). Furthermore, the dietary supplementation of MCE led to a significant enhancement in eggshell thickness and Haugh unit at wk 8 and the data showed a statistically significant linear and quadratic increase (*P* < 0.05). Serum cytokine assay showed that dietary supplementation of MCE led to linear and quadratic increases in IL-4 and IL-10 level (*P* < 0.05). Dietary supplementation of 350 and 450 mg/kg MCE was observed to result in linear and quadratic increase in serum lysozyme levels (*P* < 0.05). The addition of MCE to the diet resulted in a linear and quadratic increase in the levels of sIgA in the jejunum and ileum (*P* < 0.05). In terms of gene expression, the addition of MCE to the diet resulted in linear and quadratic increases in the expression of *IL-10, IgA, Serpinb14, Serpinb14B,* and *OIH* (*P* < 0.05). The expression of jejunal genes *pIgR* and *IL-4* was observed to increase in a linear and quadratic manner, respectively, following the dietary addition of 350 mg/kg MCE and *IL-1β* decreased in a linear manner (*P* < 0.05). Moreover, these favorable effects were maximized at medium dosage (350 mg/kg) of MCE addition, and intestinal microbial composition in the control and MCE350 groups was assessed. 350 mg/kg MCE increased the relative abundance of *Bryobacter* and *Parasutterella* and decreased the relative abundance of *Erysipelatoclostridium* in the cecum (*P* < 0.05). Spearman correlation analysis revealed that *Bryobacter, Parasutterella, Skermanella*, and *Erysipelatoclostridium* were associated with nonspecific immune functions (*P* < 0.05). In conclusion, 350 mg/kg MCE supplementation elevated the immune response, and upregulated the expression of genes related to protein production in eggs, thereby improving egg quality. These effects may be associated with changes in the microbiota, specifically *Bryobacter, Parasutterella*, and *Erysipelatoclostridium*.

## INTRODUCTION

The intestinal function is important for chickens' health, as it is the vital organ for the digestion, and absorption of nutrients, and it plays a crucial role in immune defense mechanisms (Pickard et al., 2017). The physiological function and morphology of the intestinal tract, specifically the function of the intestinal villi and crypts, are closely linked to the efficiency of nutrient absorption. (Rattanawut et al., 2018). In addition, immune cells and cytokines found in the intestinal mucosa and lamina propria form the gut immune barrier, preventing the development of intestinal inflammation.

*Macleaya cordata*, a member of the poppy family and a traditional Chinese herbal medicine, is widely distributed in southern China (Lin et al., 2018). It contains various isoquinoline alkaloids, such as sanguinarine, allocryptopine, protopine, chelerythrine, oxysanguinarine, dihydrochelerythrine, and dihydrosanguinarine (Qing et al., 2015). Sanguinarine, the primary active component derived from *Macleaya cordata*, has been approved as a feed additive in the European Union (Song et al., 2023). *Macleaya cordata* is regarded as a high-quality alternative to antibiotics (Feng et al., 2017) due to its antibacterial (Hamoud et al., 2014), anti-inflammatory (Kosina et al., 2010), and immune-enhancing properties (Khadem et al., 2014). Therefore, it has been used in combination with other plant extracts as a dietary supplement for chickens, pigs, and other animals to enhance their growth performance (Kosina et al., 2004).

Dietary supplementation with 0.6 mg/kg MCE significantly enhanced the performance of broilers, increased average daily weight gain, and reduced the feed-to-weight ratio (Liu et al., 2022). According to a different study, 1,000 mg/kg MCE reversed the effects of heat stress-induced decrease in performance and disruption of the gut microbiota (Wang et al., 2022). In another study, the addition of sanguinarine improved both cellular and humoral immunity, and reduced the population of pathogenic microbiota in the ileum (Bavarsadi et al., 2017). Similarly, dietary supplementation with 0.7 mg/kg sanguinarine improved the growth performance of yellow feathered broilers, altered serum glucose and uric acid levels, as well as improved the cecum microbiota structure, and reduced proinflammatory elements in the intestinal mucosa (Liu et al., 2020). Furthermore, a study on Xuefeng black-bone chickens demonstrated that 200 mg/kg MCE improved egg quality, increased antioxidant capacity, and enhanced immune system function (Guo et al., 2021).

Although evidence indicates that MCE (plant extract) may confer benefits to broiler growth, suppress intestinal inflammation, and modulate gut microbiota in laying hens, however, there is a paucity of research on the impact of varying doses of MCE on egg quality through gut health and microbiota modulation in Hyline brown laying hens. Therefore, the objective of this study was to evaluate the influence of MCE on production performance, egg quality, serum immune indices, and mRNA expression of immunity-related genes in the jejunum and protein production-related genes in the magnum of laying hens. The findings will provide invaluable insights for the implementation of MCE in the management of laying hens.

## MATERIALS AND METHODS

All the study procedures followed the guidelines of the Animal Care and Use Committee, and the protocols of animal experiments were reviewed and approved by the Shandong Agricultural University (Tai'an, China) Institutional Animal Care and Use Committee.

### Experimental Design

A total of 192 Hyline brown laying hens, aged 30 wk, were randomly assigned to 4 treatment groups, with 4 replicates in each group, resulting in 48 birds per group. After a 7-d acclimatization period, the experiment commenced. The control group (**CON**) was fed a basal diet, while the other groups received a basal diet supplemented with *Macleaya cordata* extract (**MCE**) at 250 mg/kg (MCE250), 350 mg/kg (MCE350), and 450 mg/kg (MCE450). The diets were formulated to meet or exceed the National Research Council (NRC) (1994) standards for all nutrients (Table 1). The feed additive used in this study contained 0.375% isoquinoline alkaloids, including 0.15% sanguinarine. The laying hens had unrestricted access to water and were fed twice daily at 8:00 am and 5:00 pm. The experiment lasted for 8 wk.

**Table 1:** Composition and nutrition levels of the basal diet (fed basis, %).

Items	Value
Ingredients (%)	
Corn	57.00
Soybean meal (46%)	24.00
Soybean oil	1.00
Limestone	9.00
Wheat middling	5.50
Dicalcium phosphate	1.00
Salt	0.30
*DL*-methionine (98%)	0.12
Lysine-HCl (98%)	0.08
Premix^1^	5.00
Total	100.00
Nutrient level^2^	
Metabolizable energy, Mcal/kg	2.70
Crude protein, %	16.43
Calcium, %	3.62
Total phosphorus, %	0.56
Methionine, %	0.40
Lysine, %	0.89
Available phosphorus, %	0.35
Cysteine+methionine, %	0.75

1 The premix provided the following per kg of the diet: iron, 60 mg; copper, 10 mg; manganese, 80 mg; zinc 80 mg; iodine 0.3 mg; vitamin A, 12,500 IU; vitamin D3, 4000 IU; vitamin K3, 2 mg; thiamine, 1 mg; riboflavin, 8.5 mg; calcium pantothenate, 50 mg; niacin acid, 32.5 mg; pyridoxine, 8 mg; folic acid, 5 mg; B12, 5 mg; choline chloride, 500 mg; phytase, 1,000 IU

2 The nutrient levels were calculated values.

### Sample Collection

The feed intake was recorded weekly on a replicate basis, and the eggs laid were counted and weighed daily. In addition, the number of defective eggs (broken or sandy) was recorded daily. The laying rate, qualified egg rate, feed-to-egg ratio, average egg weight, and average daily feed intake were subsequently calculated.

At wk 8, 2 chickens were selected from each replicate and fasted for 12 h before slaughter. Serum was extracted from the blood and stored at -20°C for further analysis. Samples of the jejunum, mucosa of the jejunum and ileum, magnum, and cecal contents were rapidly frozen in liquid nitrogen and stored at -80°C until analysis.

### Egg Quality

At wk 4 and wk 8, 3 eggs were randomly selected from each replicate, and their quality was evaluated. The eggshell color was determined using a Minolta CR-410 colorimeter. Egg weight was measured using an electronic balance, and the length and width of the eggs were measured with a vernier caliper. Eggshell thickness was measured using an eggshell thickness gauge from Robotmation (model ETG-1061, Japan). Eggshell strength was assessed using an eggshell strength tester (model EFG-0503, Robotmation, Japan). A multifunction egg quality tester (model EMT-5200, Robotmation, Japan) was used to measure the albumen height, yolk color, and Haugh units.

### Serum and Intestinal Mucosal Immune Markers

An enzyme-linked immunosorbent assay kit (Mlbio Co., Shanghai, China) was used to measure the levels of IL-1β, lysozyme, interleukin-4 (**IL-4**), interferon-gamma (**IFN-γ**), IL-17, IL-10, and tumor necrosis factor (**TNF-α**) in the serum, as well as the level of secretory immunoglobulin A (**sIgA**) in the jejunal and ileal mucosa.

### Gene Expression

At wk 8, tissues from the jejunum and oviduct were collected and placed into RNase-free cryotubes before being stored at -80°C. Subsequently, the samples were processed in 2 mL centrifuge tubes. To each tube, 100 mg of sample, an appropriate amount of steel beads, and 1 mL of TRIzol (Invitrogen Life Technologies, Carlsbad, CA) were added, and total RNA was extracted following the manufacturer's protocol. The purity of the RNA was assessed using a nucleic acid spectrophotometer (AG 22331, Eppendorf, Hamburg, Germany). Reverse transcription was carried out with RNA samples having an OD260/OD280 ratio that exceeded 1.8 using a cDNA kit (Takara Biotechnology Co., Ltd., Beijing, China). Quantitative reverse transcription-PCR was conducted on an Applied Biosystems 7500 Fast Real-Time PCR System using an SYBR Premix Ex Taq kit (Takara Biotechnology Co., Ltd., Beijing, China), following the manufacturer's instructions. Gene expression levels were calculated using the 2^−ΔΔCt^ method, as described by Livak and Schmittgen (Livak and Schmittgen, 2001), with GAPDH serving as the reference gene. The PCR amplification program consisted of an initial step at 95°C for 15 min, followed by 40 cycles of denaturation at 95°C for 10 s and annealing/extension at 60°C for 30 s. The primer sequences for the target gene and GAPDH are presented in Table 2.

**Table 2:** Primer sequences for fluorescent quantitative PCR.

Gene	Primer sequences (5’→3’)	Accession number
*GAPDH*	F : AGAACATCATCCCAGCGTCC R : CGGCAGGTCAGGTCAACAAC	NM_204305
*AMPK-α*	F : CGGAGATAAAACAGAAGCACGAG R : CGATTCAGGATCTTCACTGCAAC	DQ302133
*IFN-γ*	F : AAAGCCGCACATCAAACACA R : GCCATCAGGAAGGTTGTTTTTC	NM_205149.1
*IgA*	F : ACCACGGCTCTGACTGTACC R : CGATGGTCTCCTTCACATCA	S40610.1
*IL-1β*	F : TGGGCATCAAGGGCTACA R : CGGCCCACGTAGTAAATGAT	NM_204524.1
*IL-4*	F : GTGCCCACGCTGTGCTTAC R : AGGAAACCTCTCCCTGGATGTC	NM_001007079.1
*IL-10*	F : CGCTGTCACCGCTTCTTCA R : TCCCGTTCTCATCCATCTTCTC	AJ621614
*IL-17*	F : CTCCGATCCCTTATTCTCCTC R : AAGCGGTTGTGGTCCTCAT	AJ493595
*NF-kB*	F : TGGAGAAGGCTATGCAGCTT R : CATCCTGGACAGCAGTGAGA	NM_205134.1
*Nrf2*	F : GAGCCCATGGCCTTTCCTAT R : CACAGAGGCCCTGACTCAAA	NM 001007858.1
*TLR2*	F : ACCTTCTGCACTCTGCCATT R : TGTGAATGAAGCACCGGTAA	NM_204278.1
*TLR4*	F : GATGCATCCCCAGTCCGTG R : CCAGGGTGGTGTTTGGGATT	NM_001030693
*TNF-α*	F : CCCCTACCCTGTCCCACAA R : TGAGTACTGCGGAGGGTTCAT	AY765397.1
*SERPINB14*	F : TTCCTGGGTAGAAAGTCAGACAAAT R : GACAATGGCATTAACCAGAACCA	NM_205152.1
*SERPINB14B*	F : CAGTGATGGCAGAACGATATGAC R : TTCCAGTTGACGTTGCTGTCTT	NM_205304.1
*OIH*	F : ACCAGCGTTGCCAAAAAGC R : GCAGGCCACCACCTGTCT	NM_001030612.1

Abbreviations: AMPK-α, Adenosine 5′-monophosphate (AMP)-activated protein kinase-α; GAPDH, glyceraldehyde-3-phosphate dehydrogenase; IFN-γ, interferon-γ; IgA, immune globulin A; IL-1β, interleukin-1β; NF-κB, nuclear factor kappa-β; Nrf2, nuclear factor erythroid 2 related factor 2; TLR2, toll-like receptor 2; TNF-α, tumor necrosis factor α.

### Cecal Microbial Community Composition

Six samples of cecal contents were prepared for each treatment group. Bacterial DNA was extracted from the cecal contents using the QIAamp DNA Fecal Mini Kit (Qiagen, Valencia, CA). The concentration of the extracted DNA was determined using a NanoDrop 2000 spectrophotometer (Thermo Scientific, MA). The V3 and V4 regions of the 16S rRNA gene were amplified using the barcoded primer duo 515F/806R (515F: 5′-GTG CCA GCM GCC GCG GTA A-3′, 806R: 5′-GGA CTA CHV GGG TWT CTA AT-3′) following previously published procedures (Wang et al., 2017). After amplification, the PCR products were visualized on a 2% agarose gel and then purified using a QIAquick Gel Extraction Kit (Qiagen, Germany). 16S rDNA pyrosequencing was performed on the Illumina HiSeq2500 PE250 platform (Illumina, San Diego, CA) at Novogene Bioinformatics Technology Co., Ltd. (Beijing, China). The sequencing data were subjected to a series of processes, including quality filtering, trimming, concatenation, and merging, using FLASH (version 1.2.11) to obtain a final high-quality sequence for further analysis. Identical operational taxonomic units (**OTUs**) were classified using UPARSE (version 7.1) from high-quality sequences with a similarity of ≥ 97%. The representative sequence of the OTU was designated as a species, and subsequently, the composition of species in each sample was quantified across different taxonomic levels using the RDP Classifier (version 2.2) against the Silva 16S rRNA database (release 119) with a confidence threshold of 70%.

### Statistical Analysis

The experimental data were analyzed using one-way ANOVA with SPSS 26.0 software. The differences between the groups were analyzed using Duncan multiple comparisons, and the measured indicators were compared with linear and quadratic polynomials. When *P* < 0.05, the difference is significant. *P* < 0.01 indicates high significance, 0.05 ≤ *P* < 0.10 indicates a trend toward significance.

## RESULTS

### Laying Performance and Egg Quality

Table 3 illustrates the impact of MCE on the laying performance of hens. The dietary supplementation of MCE did not influence the laying performance. There was a linear and quadratic increase in the egg weight and Haugh unit with the MCE supplementation during the first 4 wk of the experiment (*P* < 0.05). The addition of 350 mg/kg MCE to the diets resulted in a significant increase in the Haugh unit of the eggs (*P* < 0.05). The dietary supplementation of MCE resulted in a linear and quadratic increase in eggshell strength at wk 8 of the experiment (*P* < 0.05). Furthermore, MCE supplementation increased the egg weight, eggshell thickness, and Haugh unit of the eggs (*P* < 0.05). Furthermore, the data demonstrated a statistically significant linear and quadratic increase (*P* < 0.05) (Table 4).

**Table 3:** Effects of MCE on laying performance of Hyline brown laying hens.

	Groups(MCE mg/kg)					*P-value*		
Item	0	250	350	450	SEM^1^	ANOVA	Linear	Quadratic
Laying rate, %	92.485	92.894	93.018	94.122	0.465	0.684	0.239	0.480
Qualified egg rate, %	96.291	96.383	96.351	97.086	0.348	0.864	0.470	0.706
ADEW, g/d	59.405	60.929	61.675	62.755	0.743	0.482	0.106	0.281
ADFI, g/d	117.761	117.825	118.763	119.333	0.614	0.802	0.320	0.609
FCR	1.995	1.933	1.926	1.901	1.939	0.632	0.205	0.430

1 SEM: standard error of the mean. Abbreviation: MCE, *Macleaya cordata* extract; ADEW, average daily egg weight; ADFI, average daily feed intake; FCR, feed conversion ratio.

**Table 4:** Effect of MCE on the egg quality of Hyline brown laying hens.

Item		Groups(MCE mg/kg)					*P-value*		
		0	250	350	450	SEM^1^	ANOVA	Linear	Quadratic
Wk 4									
Egg weight (g)		50.431	52.444	53.081	52.818	0.424	0.251	0.039	0.046
Egg-shaped index		1.278	1.280	1.281	1.293	0.005	0.896	0.290	0.506
Eggshell strength (N/m^2^)		39.699	40.420	41.558	41.423	0.625	0.861	0.262	0.506
Eggshell thickness (mm)		0.312	0.311	0.319	0.316	0.003	0.844	0.428	0.703
Eggshell	Luminosity (L*)	54.948	56.473	56.823	56.754	0.380	0.320	0.090	0.137
	Redness (a*)	20.909	21.054	21.089	21.074	0.148	0.959	0.692	0.893
	Yellowness (b*)	28.883	29.287	29.543	29.454	0.127	0.113	0.084	0.141
Protein height (mm)		5.531	5.950	6.038	5.900	0.080	0.607	0.096	0.053
Yolk color (Roche scale)		6.125	6.313	6.625	6.500	0.166	0.723	0.337	0.568
Haugh unit (HU)		73.619^b^	75.625^b^	81.850^a^	77.263^ab^	0.584	0.018	0.001	0.001
Wk 8									
Egg weight (g)		52.888^b^	53.000^b^	55.044^a^	55.600^a^	0.468	0.016	0.014	0.048
Egg-shaped index		1.288	1.283	1.289	1.276	0.003	0.577	0.363	0.538
Eggshell strength (N/m^2^)		38.699	42.180	44.296	43.355	0.581	0.192	0.001	0.001
Eggshell thickness (mm)		0.294^b^	0.323^a^	0.330^a^	0.326^a^	0.003	0.003	0.001	0.001
Eggshell	Luminosity (L*)	55.492	55.340	56.676	55.794	0.332	0.617	0.454	0.654
	Redness (a*)	19.718	20.186	20.859	20.003	0.169	0.165	0.315	0.086
	Yellowness (b*)	28.461	29.018	29.474	29.046	0.181	0.255	0.175	0.157
Protein height (mm)		5.769	6.086	6.231	6.350	0.106	0.285	0.046	0.123
Yolk color (Roche scale)		6.313	6.438	6.688	6.625	0.097	0.883	0.173	0.355
Haugh unit (HU)		78.494^b^	85.088^a^	87.881^a^	87.606^a^	0.763	0.046	0.001	0.001

1 SEM: standard error of the mean.

a,b Means within a row with different superscripts for each factor are significantly different (*P* ˂ 0.05). Abbreviation: MCE, *Macleaya cordata* extract.

### Serum and Intestinal Mucosal Immune Markers

**Figure 1:**
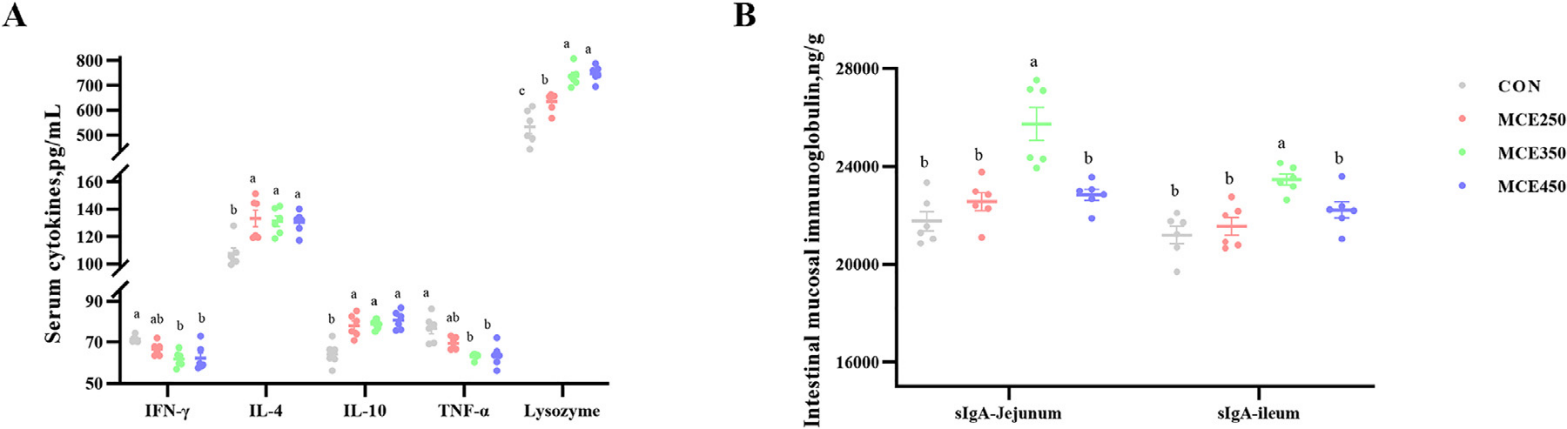
Effects of MCE on serum and jejunum-ileum mucosal immune indices in laying hens. ^a,b^ Means within a row with different superscripts for each factor are significantly different (*P* ˂ 0.05).

As shown in Table 5 and Fig. 1, serum cytokine assay indicates that dietary supplementation of MCE led to linear and quadratic increases in IL-4 and IL-10 levels (*P* < 0.05). Furthermore, the dietary supplementation of MCE increased serum concentrations of IL-4 and IL-10 in laying hens(*P* < 0.05). The addition of MCE to the diet resulted in a linear and quadratic decrease in serum IFN-γ and TNF-α levels in laying hens (*P* < 0.05). Furthermore, 350 and 450 mg/kg MCE were observed to significantly decrease serum IFN-γ and TNF-α levels (*P* < 0.05). In addition, there was an increase in serum lysozyme levels, and a linear and quadratic increase in serum lysozyme levels was observed (*P* < 0.05). The addition of MCE to the diet resulted in a linear and quadratic increase in jejunal and ileal mucosal sIgA levels (*P* < 0.05). The sIgA levels significantly increased in the jejunal and ileal mucosa of birds fed with the diet supplemented with 350 mg/kg MCE (*P* < 0.05).

**Table 5:** Regression analysis of MCE on serum and jejunum-ileum mucosal immune indices in laying hens.

	Groups(MCE mg/kg)					*P-value*		
Item	0	250	350	450	SEM^1^	ANOVA	Linear	Quadratic
IFN-γ, pg/mL	71.552^a^	66.820^ab^	61.922^b^	62.420^b^	1.107	0.001	0.001	0.001
IL-4, pg/mL	107.505^b^	133.147^a^	131.110^a^	130.297^a^	3.022	0.003	0.011	0.001
IL-10, pg/mL	64.407^b^	78.070^a^	78.812^a^	80.752^a^	1.622	0.002	0.001	0.001
TNF-α, pg/mL	76.735^a^	69.440^ab^	63.215^b^	63.672^b^	1.429	0.004	0.001	0.001
Lysozyme, pg/mL	532.917^c^	635.157^b^	736.032^a^	747.398^a^	20.133	0.001	0.001	0.001
sIgA(Jejunum),ng/g	21,760.578^b^	22,565.382^b^	25,731.728^a^	22,837.500^b^	376.960	0.001	0.056	0.003
sIgA(Ileum), ng/g	21,192.403^b^	21,546.358^b^	23,467.948^a^	22,222.238^b^	236.010	0.001	0.014	0.008

1 SEM: standard error of the mean.

a,b Means within a row with different superscripts for each factor are significantly different (*P* ˂ 0.05). Abbreviation: MCE, *Macleaya cordata* extract; IL-4, interleukin-4; IFN-γ, Interferon γ; IL-10, interleukin-10; TNF-α, tumor necrosis factor-α; sIgA, secretory immunoglobulin A.

### Gene Expression in the Jejunum and Oviduct

**Figure 2:**
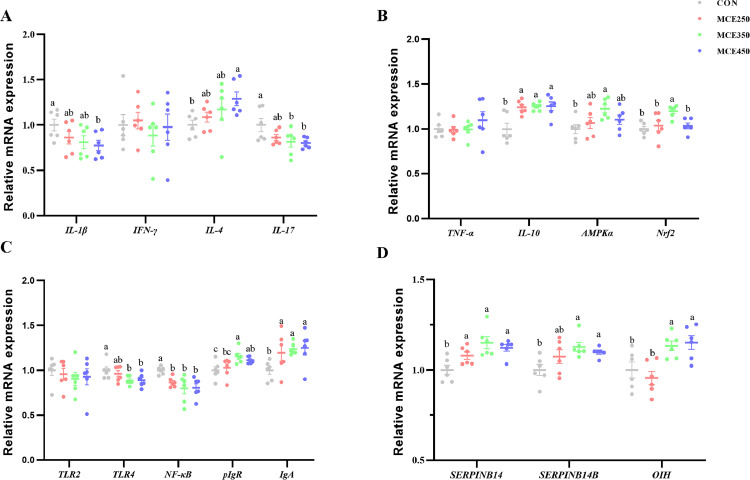
Effects of MCE on jejunal (A, B, and C) and magnum (D) gene expression in laying hens ^a,b^ Means within a row with different superscripts for each factor are significantly different (*P* ˂ 0.05).

As shown in Table 6 and Fig. 2, dietary supplementation of MCE resulted in a linear and quadratic increase in the gene expression of *IL-10, IgA,* and *Serpinb14* (*P* < 0.05). Conversely, dietary supplementation of MCE decreased the gene expression of *NF-κB*, and there was a linear and quadratic decrease in the gene expression of *NF-κB* (*P* < 0.05). Furthermore, a linear and quadratic decrease was observed in the expression of *IL-17* and *TLR4* in the jejunum (*P* < 0.05), while a linear and quadratic increase was observed in the expression of *Serpinb14B* and *OIH* in the magnum (*P* < 0.05). Notably, 350 and 450 mg/kg MCE significantly reduced the expression level of *IL-17* (*P* < 0.05), while *TLR4* showed a tendency to decrease (0.05 < *P* < 0.1). In contrast, the expression levels of *Serpinb14B* and *OIH* in the magnum were found to be significantly increased (*P* < 0.05). The dietary supplementation of MCE resulted in a linear and quadratic increase in the expression of jejunal genes *pIgR* and *IL-4* (*P* < 0.05). Similarly, *AMPKα* and *Nrf2* showed quadratic increases (*P* < 0.05), while *IL-1β* demonstrated a linear decrease (*P* < 0.05). Compared with the control group, dietary supplementation of 350 mg/kg MCE significantly increased the expression levels of *pIgR, AMPK*α and *Nrf2* (*P*<0.05), while 450 mg/kg MCE significantly increased the expression level of *IL-4* and decreased the expression level of *IL-1β* (*P* < 0.05).

**Table 6:** Regression analysis of MCE on jejunal and magnum gene expression in laying hens.

	Groups(MCE mg/kg)					*P-value*		
Item	0	250	350	450	SEM^1^	ANOVA	Linear	Quadratic
*IL-1β*	1.000^a^	0.862^ab^	0.811^ab^	0.775^b^	0.036	0.120	0.020	0.052
*IFN-γ*	1.000	1.050	0.887	0.977	0.057	0.802	0.657	0.895
*IL-4*	1.000^b^	1.087^ab^	1.172^ab^	1.290^a^	0.044	0.101	0.011	0.041
*IL-17*	1.000^a^	0.863^ab^	0.816^b^	0.802^b^	0.029	0.047	0.009	0.018
*IL-10*	1.000^b^	1.242^a^	1.251^a^	1.254^a^	0.031	0.001	0.003	0.001
*TNF-α*	1.000	0.992	0.995	1.099	0.072	0.950	0.649	0.842
*AMPKα*	1.000^b^	1.068^ab^	1.226^a^	1.104^ab^	0.030	0.041	0.075	0.050
*Nrf2*	1.000^b^	1.038^b^	1.200^a^	1.036^b^	0.024	0.008	0.221	0.045
*TLR2*	1.000	0.958	0.905	0.929	0.057	0.951	0.612	0.849
*TLR4*	1.000^a^	0.963^ab^	0.887^b^	0.891^b^	0.018	0.053	0.009	0.028
*NF-κB*	1.000^a^	0.866^b^	0.799^b^	0.806^b^	0.025	0.008	0.002	0.002
*pIgR*	1.000^c^	1.028^bc^	1.158^a^	1.114^ab^	0.021	0.015	0.009	0.021
*IgA*	1.000^b^	1.196^a^	1.238^a^	1.250^a^	0.037	0.048	0.013	0.019
*Serpinb14*	1.000^b^	1.079^a^	1.151^a^	1.122^a^	0.017	0.003	0.002	0.001
*Serpinb14B*	1.000^b^	1.073^ab^	1.128^a^	1.098^a^	0.016	0.025	0.013	0.009
*OIH*	1.000^b^	0.956^b^	1.133^a^	1.152^a^	0.025	0.002	0.002	0.007

1 SEM: standard error of the mean.

a,b Means within a row with different superscripts for each factor are significantly different (*P* ˂ 0.05). Abbreviation: MCE, Macleaya cordata extract; AMPK-α, Adenosine 5′-monophosphate (AMP)-activated protein kinase-α; IFN-γ, interferon-γ; IgA, immune globulin A; IL-1β, interleukin-1β; IL-4, interleukin-4; IL-17, interleukin-17; IL-10, interleukin-10; NF-κB, nuclear factor kappa-β; Nrf2, nuclear factor erythroid 2 related factor 2; TLR2, toll-like receptor 2; TLR4, toll-like receptor 4; TNF-α, tumor necrosis factor α; pIgR, polymeric immunoglobulin receptor.

### Cecal Microbial Community Composition

**Figure 3:**
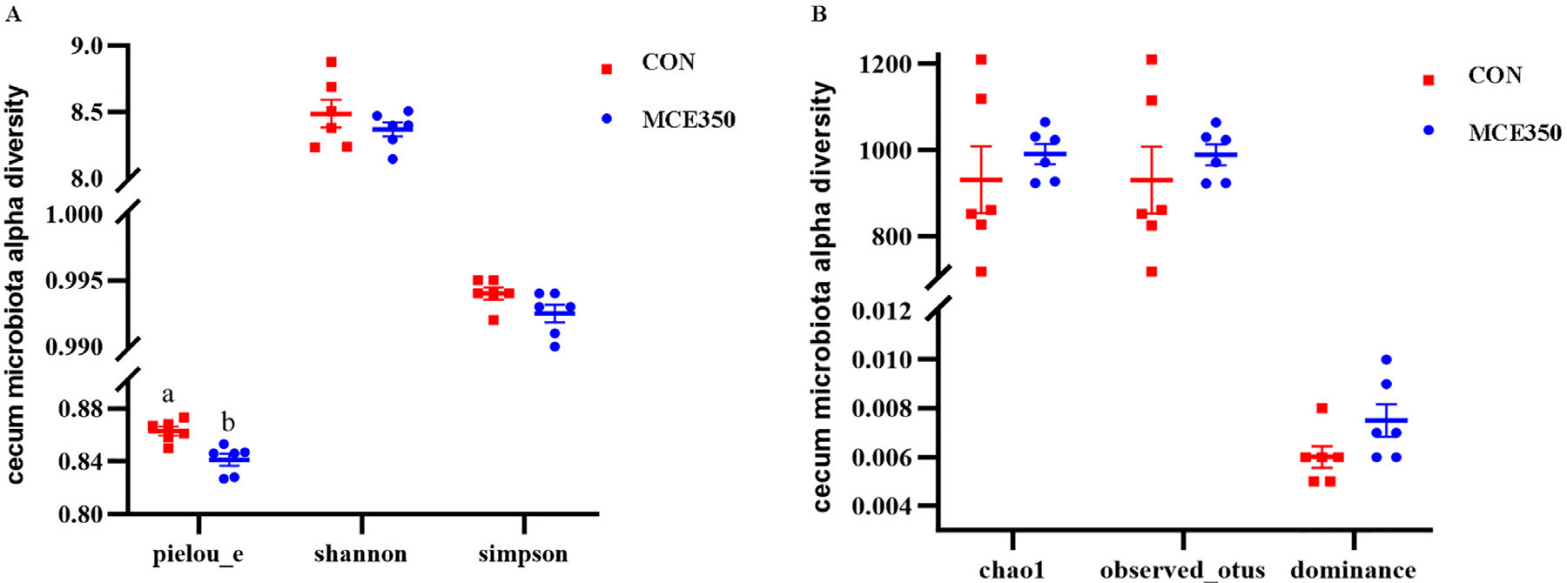
Effects of MCE on the cecal microbiota α-diversity of Hyline brown laying hens.It was analyzed using the Pielou_e index, Shannon index and Simpson index (A), chao1 index, dominance index, and observed_otus index (B). CON: control. MCE350: 350 mg/kg MCE.

**Figure 4:**
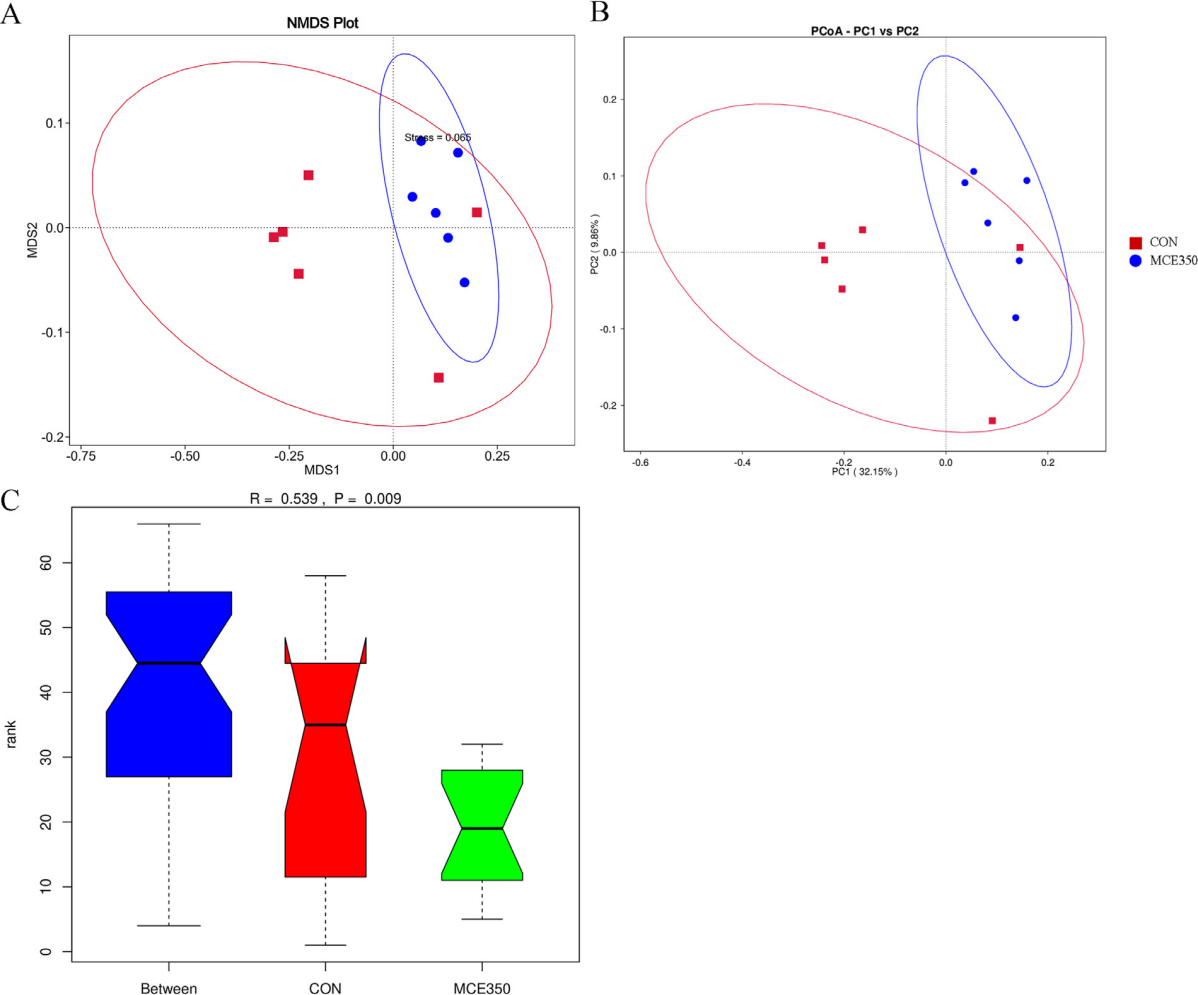
Effects of MCE on the cecal microbiota β-diversity of Hyline brown laying hens.It was analyzed by NMDS (A), PCoA (B) and Anosim (C). CON: control. MCE350: 350 mg/kg MCE.

**Figure 5:**
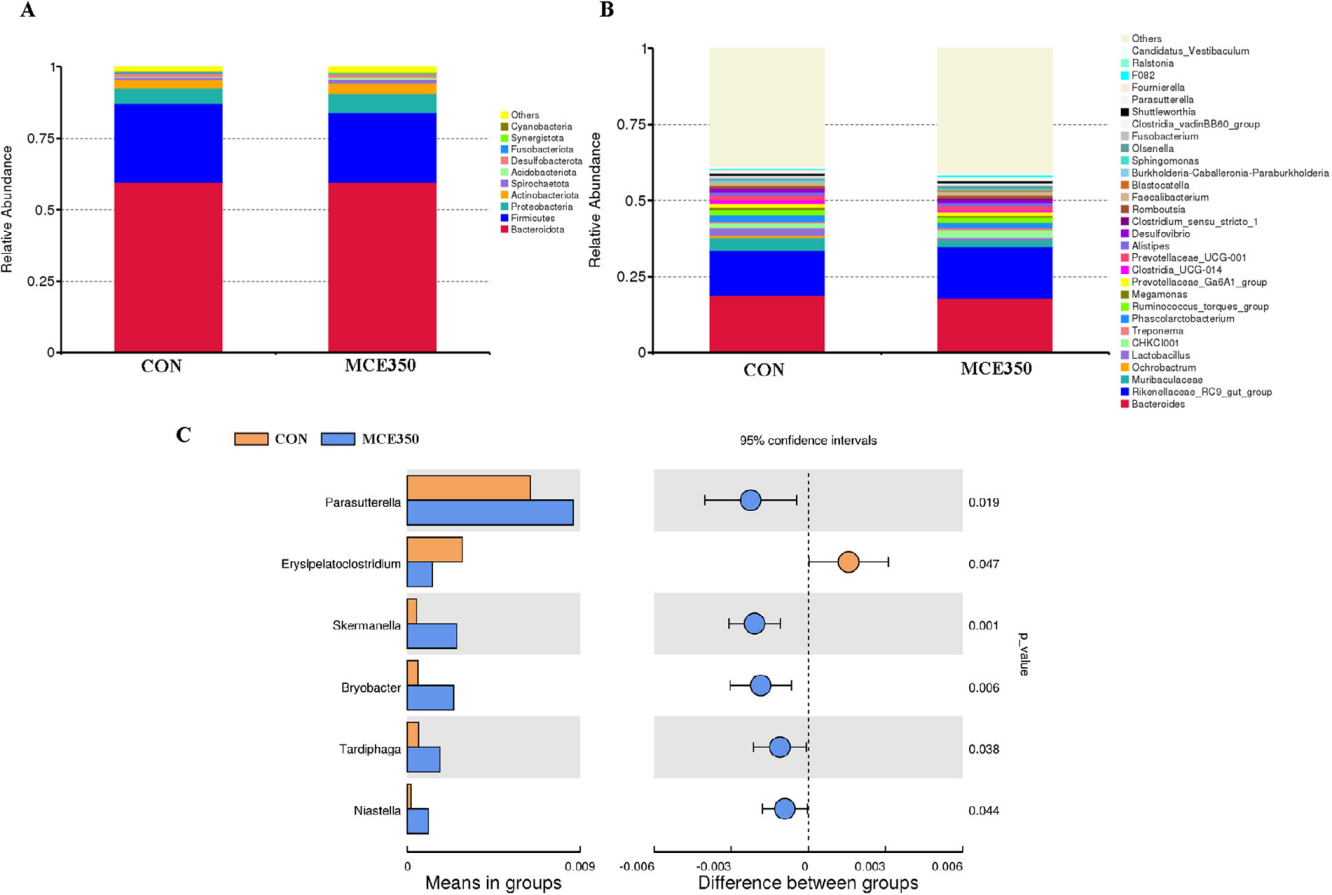
Effects of MCE on the cecal microbiota of Hyline brown laying hens. The top 10 micro-organisms at the phylum level (A) and the top 30 microorganisms at the genus level (B) in the cecum of laying hens. Differences in microorganisms in the cecum of laying hens according to the t-test (C). CON: control. MCE350: 350 mg/kg MCE.

The findings revealed that MCE influenced the alpha diversity of the cecal microbiota (Fig. 3). The dietary supplementation of MCE led to a significant reduction in the Pielou_e index in the MCE350 group (*P* ˂ 0.05), indicating an increase in dominance (*P* = 0.092) and a decrease in the Simpson index (*P* = 0.092). Nonmetric multidimensional scaling analysis (**NMDS**) and principal coordinate analysis (**PCoA**) were subsequently carried out to assess similarities and differences between the samples and groups (Figs. 4A and 4B), and statistically significant differences in microbial beta diversity were analyzed by ANOSIM (Chapman and Underwood, 1999), We found that R = 0.539 > 0, indicating that the difference between groups was greater than the difference between individuals within groups, and *P* = 0.009 < 0.01, indicating that the difference in cecum flora between the two groups was significant (Fig. 4C). The relative abundance of microorganisms in the cecal contents of Hyline brown laying hens is shown in Fig. 5A, indicating no significant difference in species richness at the phylum level between the CON and MCE350 groups. Furthermore, the relative abundance of the 30 dominant genera in each group was shown in Fig. 5B. The results showed that dietary supplementation of MCE significantly reduced the relative abundance of Muribaculaceae in the cecum but increased the relative abundances of *Clostridium_sensu_stricto_1, Sphingomonas*, and *Parasutterella*, indicating notable changes in the microbial community.

As illustrated in Fig. 5C, compared to the CON group, the relative abundance of *Parasutterella, Skermanella, Bryobacter, Tardiphaga*, and *Niastella* was significantly greater in birds in the MCE350 group. Additionally, the relative abundance of *Erysipelatoclostridium* decreased significantly in the MCE350 group (*P* < 0.05).

**Figure 6:**
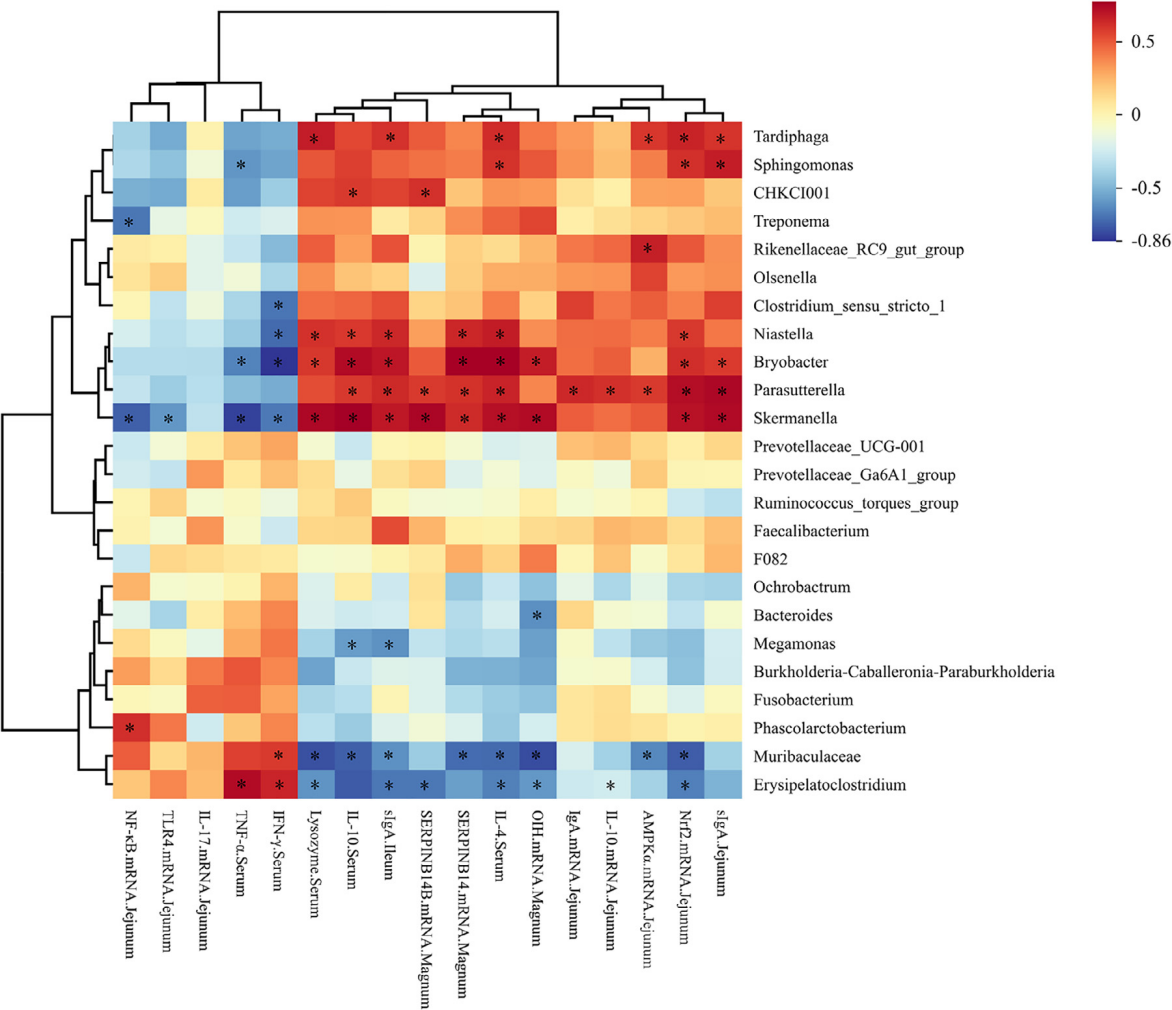
Correlation heatmap of differential microbes in the cecum and differential parameters of laying hens. Spearman's correlations were calculated for all significantly different parameters and differential cecal microbes at the genus level. The colors of squares represent the *r* values of spearman's correlation coefficient. * *P* < 0.05.

Moreover, we conducted Spearman correlation analyses on the 16S rRNA gene data to preliminarily explore possible correlations between changes in flora and changes in epigenetic indicators. As illustrated in Fig. 6, IFN-γ and TNF-α showed a negative correlation with *Skermanella, Faecalibacterium, Niastella, Bryobacter*, and *Clostridium_sensu_stricto_1* but a positive correlation with *Erysipelatoclostridium.* On the other hand, IL-4, lysozyme, and sIgA were significantly positively correlated with *Skermanella, Bryobacter, Parasutterella*, and *Tardiphaga* and significantly negatively correlated with Muribaculaceae and *Erysipelatoclostridium.* Additionally, the intestinal mucosa sIgA concentration was significantly positively correlated with the abundances of *Skermanella, Niastella, Bryobacter, Parasutterella, Tardiphaga*, and *Skermanella* but significantly negatively correlated with the abundances of Muribaculaceae, *Erysipelatoclostridium*, and *Megamonas.* The expression of the jejunal gene *Nrf2* showed a significant positive correlation with that of *Skermanella, Sphingomonas, Tardiphaga, Parasutterella, Bryobacter*, and *Niastella* and a significant negative correlation with *that of Erysipelatoclostridium* and Muribaculaceae*.* Furthermore, the gene encoding *AMPKα* exhibited a significant positive correlation with *Tardiphaga, Parasutterella*, and *Rikenellaceae_RC9_gut_group* and a significant negative correlation with Muribaculaceae. Conversely, the *SERPINB14B* gene was positively correlated with *Parasutterella, CHKCI001*, and *Skermanella* and negatively correlated with *Erysipelatoclostridium*. Similarly, *SERPINB14* showed a significant positive correlation with *Parasutterella, Bryobacter, Niastella*, and *Skermanella* and a significant negative correlation with Muribaculaceae. Finally, *OIH* exhibited a significant positive correlation with *Bryobacter* and *Skermanella* and a significant negative correlation with *Bacteroides, Erysipelatoclostridium,* and Muribaculaceae.

Overall, the Spearman correlation analysis revealed that *Erysipelatoclostridium* is associated with inflammation in the cecum microbiota, while *Parasutterella* and *Bryobacter* are associated with anti-inflammatory effects. In addition, *Parasutterella* and *Skermanella* are associated with protein production in eggs.

## DISCUSSION

### Production Performance

Plant extracts have emerged as a new category of feed additive that has gained significant popularity in poultry production in recent years. These extracts have demonstrated considerable potential in optimizing multiple facets of animal performance, such as increasing egg production efficiency, improving intestinal health, and enhancing immune system efficacy (Bojjireddy et al., 2013; Kantas et al., 2015). One such extract is derived from *Macleaya cordata* and is characterized by its primary active ingredient, sanguinarine, which has been utilized as a poultry feed additive. Recent investigations into the incorporation of MCE into feed formulations for white-feathered broilers have produced favorable results, including enhanced body weights and decreased feed-to-gain ratios (Liu et al., 2022). In the evaluation of laying hens, key performance indicators include egg production and feed conversion (Wan et al., 2021). Nevertheless, no significant alterations in laying performance were observed when MCE was added to the diets of the laying hens in this particular trial. This finding is not isolated, as Guo et al. (2021) supplemented Xuefeng black-bone chicken diets with MCE at varying concentrations of 100, 150, and 200 mg/kg (containing 7.5% sanguinarine) and similarly found no significant effect on the laying rate or average egg weight. However, Wang et al. (2022) demonstrated that the combination of 0.6 mg/kg MCE with the probiotic *Bacillus* resulted in a significant improvement in the egg production rate and average egg weight. Notably, no significant differences were observed when MCE or the probiotic *Bacillus* were administered alone. This suggests that the efficacy of MCE in enhancing the laying performance is closely linked to the dosage and concentration of MCE utilized, as well as the method and mode of its administration. Further investigations are necessary to unravel the intrinsic mechanisms underlying these observations.

Egg quality serves as a crucial determinant of profitability in the egg and poultry industry, as indicated by various studies (Geng et al., 2018; Liu et al., 2018; Ketta et al., 2020). Among the various metrics scrutinized in the market, egg weight, eggshell thickness, and Haugh units have attracted significant interest as they can affect the egg grade (Samiullah et al., 2017), resistance to breakage (De Ketelaere et al., 2002), and freshness (Bozkurt et al., 2009). Our study revealed that 350 mg/kg MCE significantly increased the Haugh unit after wk 4 of usage. Moreover, at wk 8, there was a notable increase in egg weight in the MCE350 and MCE450 groups. Furthermore, the experimental group, which received MCE supplementation, exhibited notable increases in both eggshell thickness and Haugh unit.

Extensive research has indicated that incorporating plant extracts into diets can enhance egg quality (Wang et al., 2018; Xie et al., 2019). For instance, the concurrent use of Curcuma and Scutellaria extracts improved shell thickness, egg diameter, and shell strength in Lohmann's laying hens under heat-stress conditions (Giannenas et al., 2022). Similarly, mulberry leaf extract has been found to enhance eggshell strength (Zhang et al., 2022a). Egg white, a vital component of eggs, not only provides nutrients to the embryo but also safeguards against microbial threats (Dombre et al.,2017). Ovalbumin and OVAY are the principal constituents responsible for egg white formation (Mann, 2007), while Ovo inhibitors prevent bacterial growth, preserving egg freshness and supporting embryo development (Huang et al.,2019). In this study, the addition of 350 mg/kg MCE improved the rupture resistance and freshness of the eggs. The mRNA expression of *SERPINB14, SERPINB14B,* and *OIH* was significantly increased in the MCE test group. These findings highlight how including MCE in the diet can enhance the function of the magnum in laying hens, thereby influencing egg quality. Similar to the findings of previous studies, this study indicates that the inclusion of MCE in rations does not significantly affect laying performance. However, the addition of 350 mg/kg MCE improved egg weight, eggshell thickness, and Haugh units.

### Serum Cytokine Levels, Jejunal Gene Expression, and Mucosal Immunity

In the avian innate immune system, Toll-like receptors (**TLRs**) function as recognition receptors, leading to increased expression of genes encoding inflammatory cytokines and activation of immune mechanisms (Chen et al., 2013; Kawasaki and Kawai, 2014). *TLR4* activation through lipopolysaccharide (LPS) triggers the expression of cytokines such as TNF-α and IFN-γ, which initiate innate immune responses (Płóciennikowska et al., 2015). The primary role of IFN-γ is to stimulate and regulate the immune response by promoting the Th1 response (Capobianchi et al., 2015). TNF-α, a major inflammatory cytokine (Carswell et al., 1975), plays a part in the inflammatory signaling pathway involving *NF-κB* (Pagnini et al., 2010; Qu et al., 2019). The activation of *NF-κB* by foreign xenobiotics leads to the expression of corresponding proinflammatory factors (Gordon et al., 2011).

IFN-γ and TNF-α are predominantly secreted by Th1 cells, which activate macrophages, enhance cytotoxicity, and mediate cellular immune responses. In contrast, Th2 cells are lymphocytes that play a key role in humoral immunity. Its main function is to release IL-4 and IL-10 while promoting the proliferation and production of immune cells (Hiroi et al., 1999). IL-4 acts as an anti-inflammatory cytokine, inhibiting IFN-γ activity and slowing inflammation (Gordon, 2003). Likewise, IL-10 serves as an anti-inflammatory cytokine, regulating the expression of proinflammatory cytokines and antigens to preserve the equilibrium of the inflammatory reaction (Sabat et al., 2010; Khoso et al., 2019).

The initial line of defense within the immune system is mucosal immunity, with secretory immunoglobulin A (sIgA) being a pivotal element in this mechanism. Th1 cells are believed to secrete IL-4 and TGF-β, which aid in B-cell production of IgA, resulting in increased levels of sIgA (Kunimoto et al., 1992). Lysozyme has the ability to dissolve peptidoglycan found in the bacterial cell wall. This leads to the extrusion of the bacterial structure outside of the cell due to osmotic pressure, ultimately causing bacterial death. It is particularly effective against gram-positive bacteria, which primarily consist of peptidoglycan in their cell wall (Marra et al., 2021).

In this study, the inclusion of 350 and 450 mg/kg MCE in the hens’ diet significantly decreased the serum levels of IFN-γ and TNF-α. Additionally, the MCE group exhibited significantly increased levels of IL-4, lysozyme, and sIgA in the jejunum and ileum and increased *IgA* mRNA expression. The levels of IL-10 and mRNA expression were also greater in the MCE group. This study demonstrated that the incorporation of MCE into the diet of birds increased the levels of Th2-like cytokines and secreted immunoglobulin A while improving the humoral immune function dominated by Th2 cells. This hindered the development of intestinal inflammation in broilers. These findings are consistent with those of a study conducted by Guo et al. (2021), who reported that the addition of MCE to laying hen diets decreased the serum levels of proinflammatory factors such as TNF-α and IL-10, thereby enhancing the immunity of laying hens.

Furthermore, we found that the mRNA expression level of *NF-κB* was significantly decreased in the MCE group, and the mRNA expression of jejunal *IL-17* was also down regulated in the MCE350 and MCE450 groups. This finding suggested that MCE may inhibit the *NF-κB* inflammatory pathway by reducing the expression of Toll-like receptor genes, consequently decreasing the expression of inflammatory factors. These results are consistent with the finding that the addition of 0.6 mg/kg MCE significantly down regulated hepatic *MyD88* and *NF-κB* in broiler rations, as demonstrated by Bavarsadi et al. (2017). This further supports the anti-inflammatory properties of MCE (Li et al., 2014; Wang et al., 2017).

### Gene Expression of Nrf2 and AMPKα in the Jejunum

The intestinal barrier consists of a physical barrier, a chemical barrier, an immunological barrier, and a microbiological barrier, all of which work together to maintain optimal gut health. An intact intestinal barrier is crucial for nutrient digestion and absorption, immune system function, and the balance of the gut microbiota (Di Tommaso et al., 2021). The gut, which serves as an important immune and antioxidant center, plays a significant role in combating inflammation and oxidative stress.

*Nrf2* is associated with the inhibition of oxidative stress (Sun et al., 2016). Previous studies have shown that the upregulation of *Nrf2* promotes the transcription of various antioxidant pathway proteins, with the Nrf2/HO-1 antioxidant pathway being the most common. Downregulation of Nrf2, on the other hand, leads to oxidative stress (Stockwell et al., 2017). Under normal physiological conditions, this pathway helps maintain the balance between reactive oxygen species (**ROS**) levels and the antioxidant system of organisms (Han et al., 2020). Our findings revealed a trend toward increased *Nrf2* mRNA expression in the jejunum of laying hens administered 350 mg/kg MCE. This finding is consistent with the findings of Chen et al.'s (2023) study on piglets, which demonstrated that MCE significantly increased the mRNA expression of *Nrf2* and related peroxidative disintegrative enzymes in the jejunum, suggesting that MCE can regulate the body's resistance to oxidative stress.

Studies have shown that AMPK plays a crucial role in energy metabolism and lipid synthesis in animals. The activation of AMPK promotes lipolysis and protein synthesis and provides energy to the organism. Sanguinarine has been identified as an AMPK activator (Choi et al., 2011). *AMPKα* is a key factor in vivo that influences cellular metabolism and energy regulation in organisms. In our study, 350 mg/kg MCE significantly upregulated the expression of the *AMPKα* gene in the jejunum of laying hens. This suggests that MCE may enhance nutrient metabolism, absorption, and transformation in the jejunum, providing a better energy supply for the organism. This finding aligns with the findings of Zhang et al. (2018), who reported increased *AMPKα* phosphorylation both in vitro and in vivo upon supplementation with sanguinarine.

### Cecal Microbial Community Composition

In the context of poultry farming, a robust immune system is capable of withstanding a multitude of detrimental factors, including abrupt alterations in weather patterns, deterioration of the external environment, and the onset of disease. Furthermore, it can serve to mitigate the deleterious consequences of stress. As the largest immune organ in the body, the gut not only hosts a large number of immune cells but also forms defensive barriers between various intestinal mucosal barriers through various regulatory mechanisms and signaling pathways (Sánchez et al., 2014). Moreover, it has been demonstrated that the microbial communities of the intestinal tract play an important role in maintaining normal host physiology, organ development, and metabolism (Yeoman et al., 2011; Sommer and Backhed, 2013). They have been shown to inhibit the colonization of potential pathogenic bacteria in the gastrointestinal tract of animals (Karl et al., 2017; Clavijo and Flórez, 2018). Furthermore, the cecum microbiota is involved in the maintenance of immune homeostasis and the regulation of normal immune responses (Chhabra et al., 2018).

Gut-associated lymphoid tissue (**GALT**) represents a principal site for antigen sampling and adaptive immunity induction within the gut wall (Mörbe et al., 2021). The potential of these cells to regulate intestinal immune responses during both homeostasis and inflammatory bowel disease (**IBD**) is worthy of further investigation. The findings of this study indicate that dietary supplementation with 350 mg/kg MCE may enhance immune parameters in laying hens. Accordingly, to investigate the relationship between gut microbiota and intestinal immunity, the most representative cecal microbiota and jejunal immune function indexes were selected for correlation analysis, to explore any potential correlation between changes in apparent indexes after dietary MCE supplementation and changes in cecal microbiota.

In laying hens, the cecum is mainly populated by Firmicutes and Bacteroidetes at the phylum level (Zhang et al., 2021), which are known to be closely associated with egg production performance (Singh et al., 2014). In our trial, we observed no significant differences at the phylum level between the 2 groups, and the difference in laying performance mentioned earlier was not statistically significant. The balance between gut microorganisms and their metabolites is crucial for the health of the host. Disruption of this balance can lead to metabolic disorders and dysfunctions in organisms (Hills et al., 2019). Previous reports have indicated that *Niastella* is predicted to contain several proteins with β-glucosidase activity (Bailey et al., 2013), which is an important enzyme involved in cellulose hydrolysis (Su et al., 2021). Dietary supplementation with MCE has been found to increase the presence of *Niastella* in the gut microbiota, facilitating the solubilization and absorption of nutrients such as cellulose. Bile acid metabolism has reciprocal effects on gut microorganisms (Vítek and Haluzík, 2016), and *Bryobacter* has been found to be correlated with bile acid metabolism (Zheng et al., 2022). Bile acids can influence host metabolism and immune function by regulating nutrient availability (Cai et al., 2022).

In this study, we found that an increase in the abundance of *Bryobacter* inhibits the expression of proinflammatory cytokines and facilitates the production of cytokines associated with the inhibition of inflammation development and the maintenance of egg quality. *Parasutterella* not only affects the transport and synthesis of bile acids and plays a protective role in the liver (Llopis et al., 2016; Ju et al., 2019) but also has a relationship with the inflammatory response in the intestinal mucosa (Chiodini et al., 2015). *Skermanella* species produce proteases, chitinases, and lipases (Panneerselvam et al., 2018), which work together to control pests (de Carolina Sánchez-Pérez et al., 2014). Additionally, it possesses the ability to inhibit the toxicity of the trace metal Sb (Shotyk et al., 2005), which contributes to improved tolerance to heavy metals in laying hens. Although the differences in *Tardiphaga* were significant, the role of Xanthobacteraceae as a plant-associated bacteria is uncommon in animals (Ikeda-Ohtsubo et al., 2018). *Erysipelatoclostridium*, an anaerobic bacterium, can induce inflammation and various diseases, thereby weakening the host's immune system (Zhang et al., 2022b). Research has indicated a potential connection between *Erysipelatoclostridium* and certain medical conditions, including metabolic syndrome and gout (Smith-Brown et al., 2016; Shao et al., 2017). In this study, the inclusion of MCE significantly reduced the abundance of *Erysipelatoclostridium*, and we found that MCE can eliminate and suppress harmful bacteria that induce inflammation, consequently mitigating the likelihood of intestinal inflammation.

The Spearman correlation analysis in the current study identified several cecal bacterial species, including *Tardiphaga, Sphingomonas, CHKCI001, Niastella, Bryobacter, Parasutterella*, and *Skermanella*, which were associated with the expression of genes related to the promotion of inflammation and the reduction of egg quality. The cecal bacteria that exhibited the expression of genes related to the promotion of inflammation and the reduction of egg quality were identified as *Erysipelatoclostridium*, Muribaculaceae, and *Megamonas*. Consequently, MCE markedly elevated the proportion of *Bryobacter, Parasutterella*, and *Skermanella*, which are linked to anti-inflammatory processes, while concurrently reducing the abundance of *Erysipelatoclostridium*, a pro-inflammatory bacterium, in the cecum of laying chickens. Furthermore, MCE elevated the levels of pertinent immune factors within the gut and serum, in addition to the level of immunoglobulin secreted by the intestinal mucosa. This resulted in the preservation of the intestinal environment.

## CONCLUSIONS

The inclusion of MCE in hen feed has been shown to inhibit intestinal inflammation, promote the expression of genes related to protein production in eggs, stimulate the growth of beneficial gut bacteria, reduce the presence of detrimental bacteria, and improve egg quality. These findings suggest that incorporating 350 mg/kg MCE into the diet could be valuable for laying hens feeding.

## DISCLOSURES

Chake Keerqin is employed by Phytobiotics (Jiangsu) Biotech Co. Ltd. No commercial or financial relationships with potential conflicts of interest have been reported by the other authors.

## Appendix Supplementary materials

Image, application 1
