# Supplementary material for: Macleaya cordata extract improves egg quality by altering gut health and microbiota in laying hens
Source: Poult Sci. 2024 Oct 10;103(12):104394. doi: 10.1016/j.psj.2024.104394 (PMC11538866; doi:10.1016/j.psj.2024.104394)
Supplement: Supplementary file 1 [file mmc1.pdf]

Title: *Macleaya cordata* extract improves egg quality by altering gut health and microbiota in laying hens

Manuscript Number: PSJ-D-24-00488R1

Author: Guoxin Zhang (1063507176@qq.com)

*Corresponding author. Zhigang Song (zhigangs@sdau.edu.cn)

Reasons for change of authors: In June, Guoxin Zhang, the first author, successfully completed his master's degree at Shandong Agricultural University and began his new role in the same month. The manuscript was completed during the author's studies for a master's degree at Shandong Agricultural University. The subsequent refinement and revision of the manuscript occurred at a second institution, resulting in his acquiring dual workplace identities. Consequently, the first author has added a new address.

Okasha played a crucial role in assisting me with linguistic polish and manuscript revision through online communications while I was engaged in the aforementioned revisions, including addressing questions from reviewers and editorial boards. Okasha is included among the authors as the fourth contributor, in recognition of his significant input to the paper.

Bochen Song transitioned from corresponding author to co-first author, while Zhigang Song assumed the role of corresponding author. Bochen Song was engaged in postdoctoral research in Prof. Song's laboratory, and Guoxin Zhang, the first author, was a Master of Agricultural student under Prof. Song's supervision. The research was conducted by us in collaboration. The article was authored by Guoxin Zhang, under the supervision of Bochen Song and Zhigang Song. The order of authorship has been modified to reflect the varying degrees of contribution to subsequent revisions of the article. It is beyond question that both have made a significant contribution to the work of this paper.

Author's contributions: GZ: investigation, data curation, writing original draft. BS helped with sample collection and data analyses. XP, CK and OH contributed to revising the manuscript. ZS: resources, conceptualization, methodology, funding acquisition.

After consultations, all the authors agreed with the addition of author and address in this paper; all the authors agreed with the rearrangement of the names; Below are the signatures of all authors.

Guoxin Zhang   Bochen Song   Xue Pan   Okasha H.M.

[illegible]   Zhigang Song

2024.10.1

Title: *Macleaya cordata* extract improves egg quality by altering gut health and microbiota in laying hens

Manuscript Number: PSJ-D-24-00488R1

Author: Guoxin Zhang (1063507176@qq.com)

*Corresponding author. Zhigang Song (zhigangs@sdau.edu.cn)

Reasons for change of authors: In June, Guoxin Zhang, the first author, successfully completed his master's degree at Shandong Agricultural University and began his new role in the same month. The manuscript was completed during the author's studies for a master's degree at Shandong Agricultural University. The subsequent refinement and revision of the manuscript occurred at a second institution, resulting in his acquiring dual workplace identities. Consequently, the first author has added a new address.

Okasha played a crucial role in assisting me with linguistic polish and manuscript revision through online communications while I was engaged in the aforementioned revisions, including addressing questions from reviewers and editorial boards. Okasha is included among the authors as the fourth contributor, in recognition of his significant input to the paper.

Bochen Song transitioned from corresponding author to co-first author, while Zhigang Song assumed the role of corresponding author. Bochen Song was engaged in postdoctoral research in Prof. Song's laboratory, and Guoxin Zhang, the first author, was a Master of Agricultural student under Prof. Song's supervision. The research was conducted by us in collaboration. The article was authored by Guoxin Zhang, under the supervision of Bochen Song and Zhigang Song. The order of authorship has been modified to reflect the varying degrees of contribution to subsequent revisions of the article. It is beyond question that both have made a significant contribution to the work of this paper.

Author's contributions: GZ: investigation, data curation, writing original draft. BS helped with sample collection and data analyses. XP, CK and OH contributed to revising the manuscript. ZS: resources, conceptualization, methodology, funding acquisition.

After consultations, all the authors agreed with the addition of author and address in this paper; all the authors agreed with the rearrangement of the names; Below are the signatures of all authors.

Guoxin Zhang Bochen Song Xue Pan Okasha H.M.

Chake Keerqin Zhigang Song

2024.10.1
